# Cluster size dependent coordination of formate to free manganese oxide clusters

**DOI:** 10.1039/d3cp04035f

**Published:** 2023-11-14

**Authors:** Sandra M. Lang, Thorsten M. Bernhardt, Joost M. Bakker, Robert N. Barnett, Uzi Landman

**Affiliations:** a Institute of Surface Chemistry and Catalysis, University of Ulm Albert-Einstein-Allee 47 89069 Ulm Germany sandra.lang@uni-ulm.de; b Radboud University, Institute for Molecules and Materials, FELIX Laboratory Toernooiveld 7 6525 ED Nijmegen The Netherlands; c School of Physics, Georgia Institute of Technology Atlanta GA 30332-0430 USA

## Abstract

The interaction of free manganese oxide clusters, Mn_*x*_O_*y*_^+^ (*x* = 1–9, *y* = 0–12), with formic acid was studied *via* infrared multiple-photon dissociation (IR-MPD) spectroscopy together with calculations using density functional theory (DFT). Clusters containing only one Mn atom, such as MnO_2_^+^ and MnO_4_^+^, bind formic acid as an intact molecule in both the *cis*- and *trans*-configuration. In contrast, all clusters containing two or more manganese atoms deprotonate the acid's hydroxyl group. The coordination of the resulting formate group is strongly cluster-size-dependent according to supporting DFT calculations for selected model systems. For Mn_2_O_2_^+^ the co-existence of two isomers with the formate bound in a bidentate bridging and chelating configurations, respectively, is found, whereas for Mn_2_O_4_^+^ the bidentate chelating configuration is preferred. In contrast, the bidentate bridging structure is energetically considerably more favorable for Mn_4_O_4_^+^. This binding motif stabilizes the 2D ring structure of the core of the Mn_4_O_4_^+^ cluster with respect to the 3D cubic geometry of the Mn_4_O_4_^+^ cluster core.

## Abstract 2

Manganese oxide clusters deprotonate the hydroxyl group of formic acid yielding a strongly cluster size dependent binding motif of the formate group.

## Graphical abstract

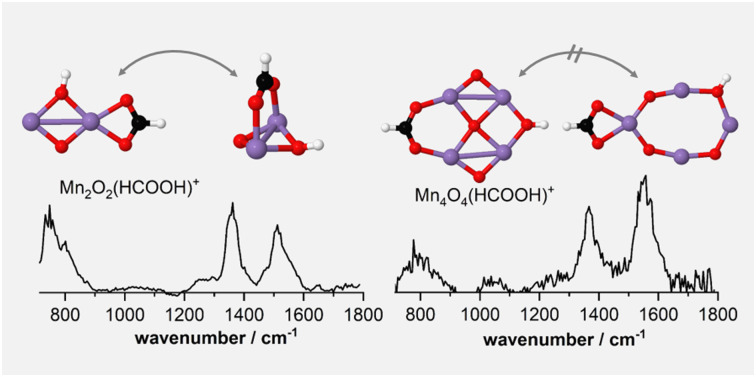

## 1 Introduction

Understanding the principles governing the oxidation of water and the rational design of novel water oxidation catalyst for sustainable (*e.g.*, solar) energy storage are major challenges in the field of alternative energy production. Such initiatives, typically called “artificial photosynthesis”,^1–5^ are often inspired by the natural oxygen evolving complex (OEC) of photosystem II. The OEC itself continues to be a subject of intense research, and has been found to comprise an inorganic CaMn_4_O_5_ cluster which is stabilized and linked to the protein backbone by several amino acid residues.^6^ Some of these amino acids, such as glutamic acid or aspartic acid, bind to the cluster by bridging two Mn (or Ca–Mn) atoms with the deprotonated carboxyl group (bridging μ-oxo ligands).

The natural inorganic cluster of the OEC has inspired significant efforts aiming at the synthesis of biomimetic bi- and tetra-nuclear manganese oxide as well as calcium–manganese oxide complexes.^7–17^ For forming and stabilizing such clusters a large number of different, sometimes complex, O- and N-donor ligands, often forming five- or six-membered rings with the metal atoms, have been reported.^8,15^ In some cases, small carboxylic acid residues, such as acetate or pivalate ligands, have been used (see *e.g.* ref. 16,18,19).

To probe fundamental aspects of the water oxidation reaction, an increasing number of research groups study small metal oxide clusters isolated in the gas phase to model water activation and oxidation catalysts.^20–26^ In our project, we investigate gas-phase clusters consisting of elements also contained in the natural OEC, *i.e.*, Mn, Ca, and O. In the first steps of this approach, we have prepared bare, non-ligated manganese^27–30^ and calcium–manganese oxide clusters^31–33^ of different size and composition, and studied their water activation and oxidation properties *via* reactivity measurements and *via* infrared spectroscopy. We now aim to increase the model system's complexity by attaching small carboxylic acids mimicking a ligand environment that could potentially stabilize the cluster structure and/or serve as proton/electron transfer media. Carboxylic acids represent the simplest mimics of the carboxyl group of amino acids and are thus suitable initial molecules for such studies.

In this contribution, we present IR-MPD spectra of a series of manganese oxide clusters containing up to nine Mn atoms ligated with one formic acid (HCOOH) molecule. We show that all clusters containing more than one Mn atom deprotonate formic acid. However, the binding of the resulting formate group is strongly cluster-size-dependent and can differ from the bridging μ-oxo configuration typically found for Mn_2_O_2_- and Mn_4_O_4_-based molecular catalysts. The consequences of cluster fluxionality^32–34^ and differences from the ligation with acetic acid^35,36^ are discussed.

## 2 Methods

### 2.1 Experimental methods

Cationic manganese oxide clusters were produced by pulsed laser ablation of a rotating manganese target using the second harmonic of a Nd:YAG laser. The ablation took place in a 3 mm diameter and 60 mm long growth channel in the presence of a short pulse of helium carrier gas seeded with 0.25% oxygen. To form cluster-HCOOH complexes, a mixture of about 0.2% HCOOH in helium was introduced *via* a second pulsed valve 50 mm downstream in a flow tube reactor (3 mm diameter, 45 mm long).

The reaction mixture was then expanded into vacuum forming a molecular beam before entering the intracavity region where it was irradiated by the IR laser beam of the Free Electron Laser for Intra Cavity Experiments (FELICE, 710–1800 cm^−1^; 10 μs pulse duration; spectral width set to approximately 0.4% FWHM of the central frequency) crossing it at an angle of 35°. A few μs after the interaction with FELICE, all clusters were extracted into a reflectron time-of-flight mass spectrometer by a set of pulsed high voltage plates and detected with a microchannel plate detector.^37,38^

To correct for long-term source fluctuations, the experiment was operated at twice the FELICE repetition rate, allowing for the recording of reference mass spectra in between successive FELICE pulses. Whenever FELICE was in resonance with an IR active vibrational mode of a given cluster, multiple IR photons were absorbed sequentially, leading to heating of the complex and finally to its fragmentation. The IR-MPD spectra shown in this contribution represent the depletion yield *Y*(*ν̃*) at wavenumber *ν̃*, calculated as 
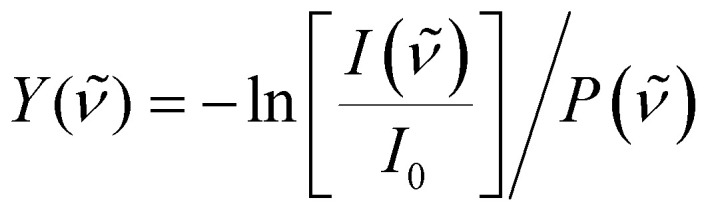
, where *I*(*ν̃*) and *I*_0_ are the mass peak intensities with and without laser light, respectively, and *P*(*ν̃*) the macropulse energy. To reduce the IR fluence with which the complexes are irradiated, the whole instrument can be translated up to 300 mm from the focus position leading to a 30-fold reduction in intensity but increased overlap between the laser and molecular beam and thus an increased signal to noise ratio. All spectra presented in this work were recorded 290 mm from the focus. To further reduce the IR fluence and increase the spectral resolution, the overlap between laser and molecular beams can be purposely misaligned such that the molecular beam only observed the lower intensity part of the laser beam.

### 2.2 Theoretical methods

The atomic arrangements of the model complexes were explored by employing the Born–Oppenheimer spin density-functional theory molecular dynamics (BO-SDFT-MD)^39^ method with norm-conserving pseudopotentials^43^ and the generalized gradient approximation (GGA) for electronic exchange and correlations, using the Perdew, Burke, Ernzerhof (PBE)^44^ functional. We have used a plane-wave basis with a kinetic energy cutoff *E*_c_ = 62 Ry (843.6 eV), which shows convergent results. This corresponds to a real-space grid spacing of 0.4*a*_0_ (with *a*_0_ the Bohr radius); the real-space grid spacing for the density (and potential) was 0.133*a*_0_ corresponding to *E*_c_ = 555 Ry.

In the construction of the Mn pseudopotentials the valence electrons, 3d^5^ and 4s^2^, were characterized by core radii *r*_c_(s) = 2.35*a*_0_ and *r*_c_(d) = 2.35*a*_0_, with the s orbital treated as local. The Mn pseudopotential had a non-linear core correction with ∼10% of the [Ar] core charge. For the oxygen atom pseudopotential the valence 2s^2^ and 2p^4^ electrons were treated with *r*_c_(s) = *r*_c_(p) = 1.45*a*_0_, with the p orbital treated as local. The BO-SDF-MD method is particularly suitable for investigations of charged systems since it does not employ a supercell (*i.e.*, no periodic replication of the ionic system is used). In all calculations, the dependence on spin multiplicity has been checked and the results that we report correspond to the spin multiplicities with the lowest energies. In the following we denote the difference between the number of spin-up and spin-down electrons by *μ* = *N*_↑_ − *N*_↓_.

The vibrational eigenmode frequencies and atomic displacement eigenvectors were determined in the harmonic approximation by direct diagonalization of the dynamical matrix, constructed by finite differencing of the forces at different points near the equilibrium geometry.^45^ From the DFT calculations for the displacements we also evaluate for each of the atoms the effective-charge tensor (the derivative of the dipole of the system with respect to the displacement of the atom), which together with the eigenmode vectors yields the oscillator strengths of the vibrational modes and the IR intensities.^46^ To correct for the general slight mismatch between the experimental and calculated vibrational frequencies, we applied an empirical scaling factor of 1.054 to all calculated vibrational modes. This factor is based on a comparison of the experimental and calculated bands I and II (isomers 2,2-A, 2,2-B, 2,4-A, 4,4-A) shown in Fig. 3–5.

## 3 Results and discussion

### 3.1 Deprotonation of formic acid on Mn_*x*_O_*y*_^+^ (*x* ≥ 2)

**Fig. 1:**
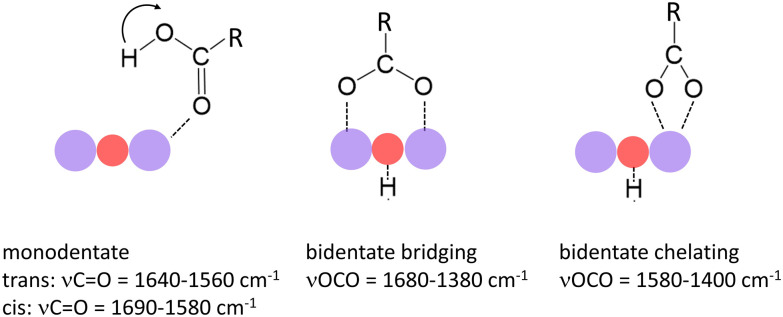
Binding modes of small carboxylic acids to manganese oxide clusters and most characteristic vibrational modes.^36,40–42^ Purple and red balls indicate Mn and O atoms of the cluster. The left mode indicates the *trans*-configuration; the arrow indicates the position of the H atom in the *cis*-configuration.

In a previous study we have shown that small carboxylic acids such as formic, acetic, or propionic acid, bind to manganese cations *via* the oxygen atom of the carboxyl group resulting in a monodentate structure with the acid in both the *trans*- (*cf.* left structure of Fig. 1) and the *cis*-configuration (indicated by the arrow).^47^ In contrast, acetic acid was shown to dissociate (deprotonate) on some manganese oxide clusters forming a hydroxyl and an acetate group, the latter typically in a bidentate bridging or chelating structure (*cf.* middle and right structure in Fig. 1).^36^ In case of molecular binding, the C=O stretch mode (*ν*C=O) of the carboxyl group is most characteristic (typically between 1690 cm^−1^ and 1560 cm^−1^) and even allows to distinguish between the *trans*- and *cis*-configuration.^36,47^ For deprotonated acids, *ν*C=O is no longer observed; instead, the symmetric and asymmetric stretch modes of the OCO group (*ν*_s_OCO and *ν*_as_OCO) become the diagnostic modes (*ν*_s_OCO typically between 1480 cm^−1^ and 1380 cm^−1^; *ν*_as_OCO typically between 1680 cm^−1^ and 1500 cm^−1^).^40–42^

**Fig. 2:**
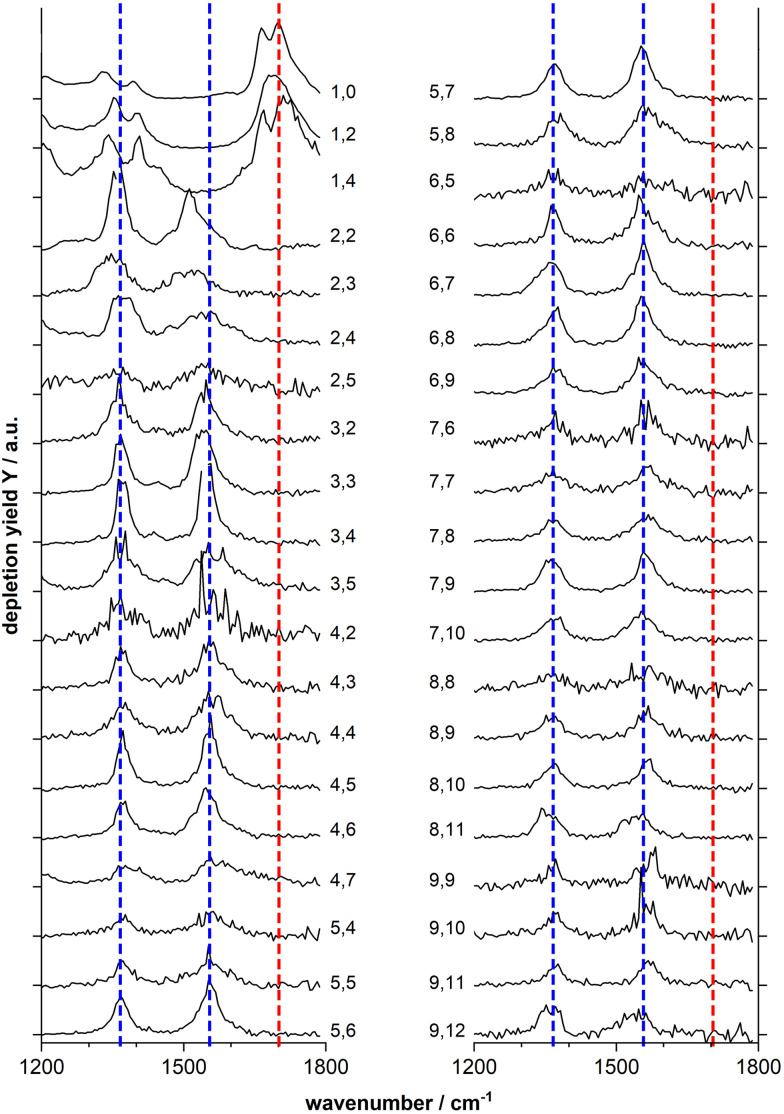
IR-MPD spectra of Mn_*x*_O_*y*_(HCOOH)^+^ complexes (labeled *x*, *y* to the right and left of the spectra, respectively). The blue and red dotted line are drawn to guide the eye. The black tick marks on the *y*-axis indicate the zero depletion yield for each spectrum.

Fig. 2 shows the IR-MPD spectra of series of Mn_*x*_O_*y*_(HCOOH)^+^ (*x* = 1–9) complexes in the 1200–1800 cm^−1^ spectral region which is most diagnostic for the different adsorption configurations. From this figure it is evident that the spectra of the mono-manganese complexes Mn(HCOOH)^+^, MnO_2_(HCOOH)^+^ and MnO_4_(HCOOH)^+^ are similar, showing a broad, and sometimes structured, band around 1700 cm^−1^, whereas the spectra of all clusters with *x* ≥ 2 are dominated by two main bands centered around 1550 cm^−1^ and 1370 cm^−1^, respectively. It should be noted that the first band is slightly red-shifted to around 1510 cm^−1^ for Mn_2_O_2_(HCOOH)^+^ and Mn_2_O_3_(HCOOH)^+^.

The double peak of Mn(HCOOH)^+^ around 1700 cm^−1^ can be assigned to *ν*C=O of intact molecularly bound HCOOH in the *trans* and *cis*-configurations, respectively.^47^ The co-existence of these two isomers appears to be maintained upon co-adsorption of one and two oxygen atoms (the two peaks are not resolved for MnO_2_(HCOOH)^+^, but the peak width indicates the overlap of two modes). This strong band clearly disappears for all other cluster sizes indicating the deprotonation of the carboxyl group. To gain more insight into the binding motif (bidentate bridging *vs.* chelating) we will in the following analyze the spectra of selected cluster sizes in more detail.

### 3.2 Mn_2_O_2_(HCOOH)^+^: bidentate chelating and bridging structures

**Fig. 3:**
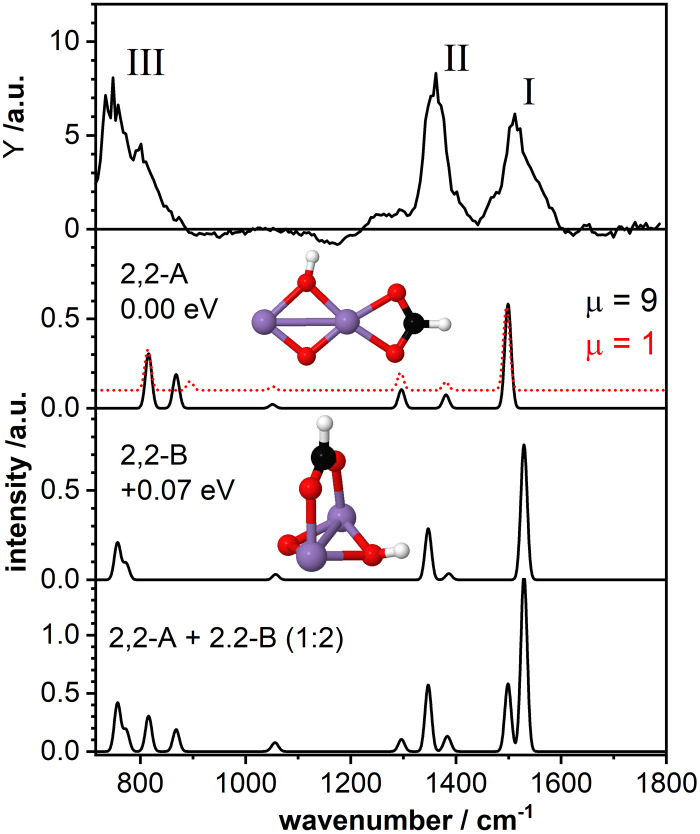
IR-MPD spectrum (top row) of Mn_2_O_2_(HCOOH)^+^ together with calculated vibrational spectra of two isomeric structures containing a formate group. For isomer 2,2-A the spectrum for the (ground-state) high spin (*μ* = 9, black curve), and for a higher-energy (by 0.09 eV compared to the low-spin ground-state) low spin isomer (*μ* = 1, red dashed curve) are shown in the second panel from the top. For isomer 2,2-B the spectrum of the (ground-state) high spin state (*μ* = 9) is shown in the third panel. The additional spectrum shown in the bottom panel represents the weighted sum of the spectra of isomer A and B (summed with a ratio of 1 : 2). Mn, O, C, and H atoms are depicted as purple, red, black, and white spheres.

The top panel of Fig. 3 displays the IR-MPD spectrum of Mn_2_O_2_(HCOOH)^+^ in the whole investigated spectral region (710–1800 cm^−1^) together with the simulated vibrational spectra for two isomeric structures. These structures contain deprotonated formic acid with the formate group in a bidentate chelating (isomer 2,2-A; Δ*E* = −2.43 eV with respect to the reactants, *μ* = 9) and bidentate bridging (isomer 2,2-B, +0.07 eV, *μ* = 9) configuration, respectively. The IR-MPD spectrum shows two bands centered at 1517 cm^−1^ (labeled I) and 1360 cm^−1^ (labeled II), both potentially broadened on the red edge, and a broad structured band between 700 and 900 cm^−1^ (labeled III).

The calculated vibrational spectrum of isomer 2,2-A exhibits a strong mode at 1499 cm^−1^ (*ν*_as_OCO), two less intense modes at 1381 cm^−1^ (*ν*_s_OCO) and 1296 cm^−1^ (CH wagging), a low intensity mode at 1051 cm^−1^ (CH out-of-plane bending) as well as two modes at 868 cm^−1^ (OH wagging) and 816 cm^−1^ (OCO bending). *ν*_as_OCO seems a plausible candidate for band I, and the modes at 868/816 cm^−1^ might explain band III, although the former, despite the scaling factor of 1.054, still lies 18 cm^−1^ below the experimental band maximum. However, the low intensity modes at 1381/1296 cm^−1^ cannot satisfactorily account for band II. From our calculations we observe a rather small energy difference (0.09 eV) between the high spin state (ground state) and the lower spin (*μ* = 1) isomer. Furthermore, the structures of these two spin states are found to be very similar (imperceptible difference on the scale of the figure (see second panel of Fig. 3)). This leads to highly similar vibrational spectra for the two isomers (black and red curves) showing that the vibrational frequencies are rather insensitive to the spin state for these system.

For isomer 2,2-B (+0.07 eV for *μ* = 9 and +0.25 eV for *μ* = 1) the *ν*_as_OCO stretch is predicted at 1529 cm^−1^ and *ν*_as_OCO and the CH bending are shifting closer together. Furthermore, the OH wagging and the OCO bending vibrations red-shift to the edge of the investigated spectral region providing a better match with the maximum of the experimental band III. Thus, bands I, II, and III are better described by isomer 2,2-B, but that cannot completely account for the overall width of band III. So, neither isomer 2,2-A nor 2,2-B give a perfect match with the experimental spectrum. The situation improves when considering the co-existence of both isomers. The bottom panel of Fig. 3 displays the weighted sum of the spectra of isomer 2,2-A and 2,2-B (summed with a ratio of 1 : 2). It should be noted that this ratio is rather randomly chosen since the relative peak intensities do not seem to allow for a quantitative determination of the contribution of both isomers. However, the summed spectrum seems to explain all experimentally observed features, even the potential broadening of bands II and III. The only mode that is not observed in the IR-MPD spectrum is the low intensity mode predicted at 1051 cm^−1^ for isomer 2,2-A and 1057 cm^−1^ for isomer 2,2-B. The lack of this mode can be attributed to its very low intensity. Thus, we conclude that the IR-MPD spectrum of Mn_2_O_2_(HCOOH)^+^ is best described by the co-existence of isomers 2,2-A and 2,2-B, which is plausible given the small energy difference between them.

### 3.3 Mn_2_O_4_(HCOOH)^+^: bidentate chelating structure

**Fig. 4:**
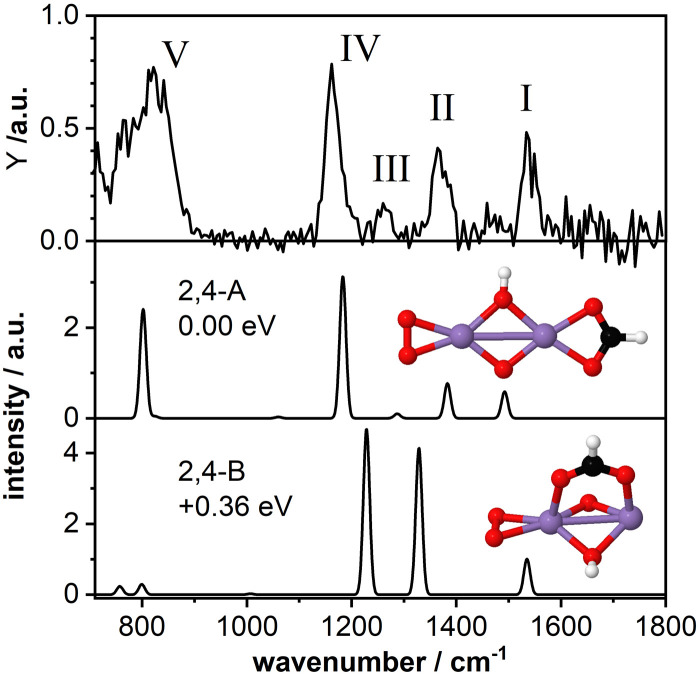
IR-MPD spectrum (top) of Mn_2_O_4_(HCOOH)^+^ together with calculated vibrational spectra of two isomeric structures containing a formate group. Mn, O, C, and H atoms are depicted as purple, red, black, and white spheres. The IR-MPD spectrum has been obtained with reduced IR macropulse fluence to achieve better resolution. The spectrum obtained at higher fluence is shown in Fig. 2.

As a second model system we have theoretically studied the complex Mn_2_O_4_(HCOOH)^+^ in more detail. The IR-MPD spectrum (Fig. 4; compared to Fig. 2 this spectrum is obtained at reduced IR macropulse fluence) shows five bands centered around 1538 cm^−1^ (labeled I), 1369 cm^−1^ (II), 1261 cm^−1^ (III), 1164 cm^−1^ (IV), and 810 cm^−1^ (V). The calculated vibrational spectrum of isomer 2,4-A (Δ*E* = −3.27 eV with respect to the reactants for *μ* = 7; +0.15 eV for *μ* = 1), with the formate in a bidentate chelating configuration, exhibits four intense modes at 1492 cm^−1^ (*ν*_as_OCO), 1383 cm^−1^ (*ν*_s_OCO), 1183 cm^−1^ (O–O stretch), and 802 cm^−1^ (a combined OH wagging and OCO bending motion) as well as a low intensity mode at 1287 cm^−1^ (CH wagging). These modes can account for all the experimentally observed bands, even for the low intensity band III. However, similar to Mn_2_O_2_(HCOOH)^+^, band I (*ν*_as_OCO) for Mn_2_O_4_(HCOOH)^+^ is predicted too low in frequency by about 46 cm^−1^ despite the scaling factor of 1.054. Also, it could be argued that only the mode predicted at 802 cm^−1^ is not sufficient to explain the broad band V.

A second isomer 2,4-B (*μ* = 7) with the formate in a bidentate bridging position is 0.36 eV higher in energy and is clearly not providing a better match with the IR-MPD spectrum. In particular, the blue-shift of the O–O stretch mode to 1229 cm^−1^ caused by the rotation of the O_2_ molecule out of the plane of the Mn_2_O_2_ cluster core breaks the agreement with band IV. Furthermore, *ν*_s_OCO is considerably red-shifted to 1329 cm^−1^, no longer matching band II very well. Finally, bands III (weak) and V (very intense) are not reproduced by this isomer. Thus, we conclude, that the IR-MPD spectrum of Mn_2_O_4_(HCOOH)^+^ best matches that of isomer 2,4-A with the formate in a bidentate chelating configuration.

### 3.4 Stabilization of the 2D Mn_4_O_4_^+^ cluster core

As a last model system, we theoretically studied the tetra-manganese complex Mn_4_O_4_(HCOOH)^+^. We have previously predicted that the bare Mn_4_O_4_^+^ cluster has a 2D ring-like structure which can gradually change to a 3D cubic geometry upon adsorption of multiple water molecules^29,30^ due to the high fluxionality of this cluster.^27,32^ The cubic structure of the bare cluster was predicted to be 0.25 eV higher in energy. Here we aim to gain insight into the structural changes of the cluster upon dissociative adsorption of formic acid.

**Fig. 5:**
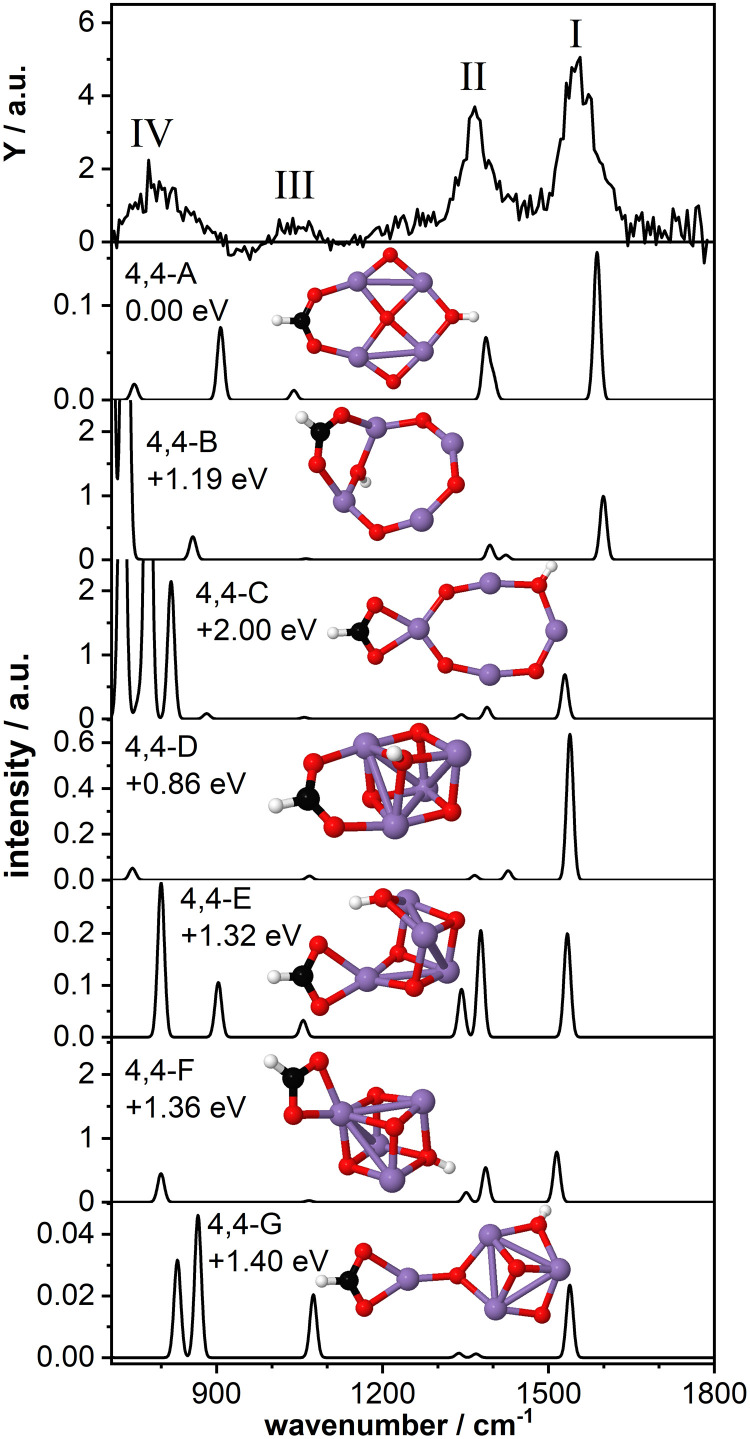
IR-MPD spectrum (top row) of Mn_4_O_4_(HCOOH)^+^ together with calculated vibrational spectra of several 2D and 3D isomeric structures. Mn, O, C, and H atoms are depicted as purple, red, black, and white spheres.

Fig. 5 shows the IR-MPD spectrum of Mn_4_O_4_(HCOOH)^+^ together with the calculated vibrational spectra of several isomers containing a ring-like and a cubic-like structure. The spectrum is similar to that for Mn_2_O_2_(HCOOH)^+^ (Fig. 3) and shows three intense bands centered around 1554 cm^−1^ (I), 1369 cm^−1^ (II), and 800 cm^−1^ (IV). In addition, a low intensity band is observed around 1046 cm^−1^ (III), close to where a band was theoretically predicted for Mn_2_O_2_(HCOOH)^+^ but not experimentally observed.

The lowest energy isomer containing deprotonated formic acid (isomer 4,4-A, *μ* = 19) found in our theoretical structure search is based on a ring-like Mn_4_O_4_^+^ cluster core. The resulting formate group bridges two Mn atoms and the hydrogen has migrated to the bridging oxygen atom opposite the formate. The bridge-binding of the formate induces the relocation of the oxygen atom initially bridging the two Mn atoms to the middle of the Mn_4_ square. In contrast, transfer of the hydrogen to the oxygen atom originally bridging the two Mn atoms where the formate has adsorbed (isomer 4,4-B, *μ* = 19) prevents the oxygen atom from being pushed inside the Mn_4_ ring. Instead, the formate group rotates out of the Mn_4_O_4_ plane allowing the hydroxyl to bridge the same Mn atoms, but opposite the formate. When the formate binds in a bidentate chelating configuration (isomer 4,4-C, *μ* = 19) the ring-like structure is also retained although it is considerably distorted. Isomers 4,4-B and 4,4-C are considerably higher in energy than isomer 4,4-A.

The vibrational spectrum of isomer 4,4-A shows two strong bands at 1588 cm^−1^ (*ν*_as_OCO) and 1400/1386 cm^−1^ (*ν*_s_OCO and CH bending) which could account for bands I and II. If so, theory overestimates the frequency of the OCO vibrations, which might indicate that the chosen scaling factor of 1.054 is too large for the Mn_4_O_4_ complex. In addition, a mode at 1040 cm^−1^ is predicted, which is in agreement with band III. The broader band IV is not so well matched by modes at 907 cm^−1^ and 751 cm^−1^. The vibrational spectrum of isomer 4,4-B is similar to that of isomer 4,4-A and could equally well explain bands I and III (with the frequency of band I even higher). However, the relative intensity of band II to band I seems to give this spectrum a poorer match with the experiment. On the other hand, the low frequency part may be a better match although the intensity of modes predicted below 800 cm^−1^ seems at odds with the rather low intensity band IV. Although this isomer is 1.19 eV higher in energy than isomer 4,4-A, both are thermodynamically accessible at −3.33 eV and −2.14 eV with respect to the reactants, respectively.

The same holds for isomer 4,4-C at 2.0 eV above isomer 4,4-A. Its spectrum contains a *ν*_as_OCO that is red-shifted to 1530 cm^−1^, now slightly lower than band I. This discrepancy is similar to the case of Mn_2_O_2_(HCOOH)^+^ discussed above. Again, the two low intensity bands around 1400 cm^−1^ do not appear a perfect match for band II. Interestingly, the more intense modes predicted below 850 cm^−1^ do match the overall width of band IV, but we hesitate about the predicted strengths.

Finally, we briefly discuss the binding of formic acid to the cubic isomer of Mn_4_O_4_^+^. In this case, we also find that bridge-binding of the formate is energetically more favorable than a chelating geometry. Most remarkably, the lowest energy cubic isomer that we found (isomer 4,4-D, *μ* = 19) is 0.86 eV higher in energy than isomer 4,4-A, although the cubic structure of the bare Mn_4_O_4_^+^ is only 0.25 eV less stable than the ring structure. This shows that adsorption of one formic acid molecule in a bridging configuration considerably stabilizes the 2D ring structure. In contrast, binding of the formate in a bidentate chelating geometry is energetically more favorable for a 3D cubic cluster core than a 2D ring-shaped cluster (compare isomer 4,4-C with isomers 4,4-E,F,G, all *μ* = 19). Nevertheless, all isomers formed with a cubic cluster core are considerably less stable than isomer 4,4-A.

For all isomers 4,4-D,E,F,G, containing a cubic cluster core, *ν*_as_OCO is, like for 4,4-C, slightly lower than for 4,4-A and 4,4-B. None of the calculated spectra provide an excellent match with the IR-MPD spectrum. For example, the low intensity modes around 1300–1400 cm^−1^ of isomers 4,4-D and 4,4-G cannot account for the intensity of band II, whereas the two more intense modes of isomers 4,4-E and 4,4-F in the same spectral range should probably have led to a splitting or at least a considerable broadening of mode II. Although they all provide a plausible match for band III, none has a convincing match for band IV, but so neither have any of the spectra calculated for 4, 4-A and 4,4-B, or 4,4-C.

Consequently, we base our assignment predominantly on the comparison for bands I-III and find the overlap with isomer 4,4-A the most plausible. We conclude that formic acid readily deprotonates on Mn_4_O_4_^+^ most likely binding formate in a bidentate bridging geometry on a ring-shaped Mn_4_O_4_^+^ cluster core (isomer 4,4-A).

### 3.5 Carboxylic acids as ligands for MnO based cluster catalysts

The IR-MPD spectra of Mn_*x*_O_*y*_(HCOOH)^+^ revealed the deprotonation of the hydroxyl group for all clusters with *x* ≥ 2, whereas formic acid is absorbed as intact molecule on Mn^+^, MnO_2_^+^, and MnO_4_^+^. Similar results were previously found for acetic acid.^36^ In contrast, for Mn_2_O_4_^+^ and Mn_3_O_5_^+^ indications of an intact acetic acid were found, suggesting deprotonation is kinetically hindered for these cluster sizes. This difference between formic and acetic acid can likely be attributed to the former's higher acidity.

More important is, however, the binding motif of the resulting formate and acetate groups, respectively. For both Mn_2_O_2_^+^ and Mn_2_O_4_^+^ binding of formate and acetate^36^ in the bidentate chelating configuration is energetically more favorable which is in agreement with the IR-MPD spectra. Only formate can also bind to Mn_2_O_2_^+^ in a bidentate bridging structure. This indicates that bridge-binding of a carboxylic acid residue on a bare di-manganese oxide cluster core is generally rather unfavorable. This seems to conflict with the reported bridge-bound acetate (OAc) groups of molecules synthesized in the liquid phase. However, in all these cases the Mn atoms were co-coordinated to larger ligands, which can potentially block the chelating position (see *e.g.* structures 51 to 67 in ref. 8 or structures 295 and 297 in ref. 15).

The situation changes for the Mn_4_O_4_^+^ cluster. The lowest energy structure, to which we assign the IR-MPD spectrum, is a 2D ring-like structure, which is stabilized by bridge-binding of a formate group. Additionally, for a 3D cubic core the bridging structure is energetically more favorable than the chelating configuration. So far, only few cubic Mn_4_O_4_ clusters with carboxylic residues as stabilizing ligands have been reported; among these are Mn_4_O_4_ cores stabilized on vanadium-^18^ and tungsten-^48^ based polyoxometalates (POMs), respectively, capped with three acetate groups as well as a Mn_4_O_4_ cubic core with three bridge bound acetate groups besides other ligands.^17^ In all these examples most of the Mn atoms are either co-coordinated to the POM or other ligands, which might potentially inhibit chelating of the acetate. However, with our free cluster model system we show that bridge-binding of a first formate group is considerably more stable even without additional stabilizing ligands. Furthermore, while the Mn_2_O_2_^+^ and Mn_2_O_4_^+^ cluster cores appear to be rather unaffected by formate binding, it induces considerable structural changes to the Mn_4_O_4_^+^ core. The particular structural fluxionality of Mn_4_O_4_^+^ has previously been described with respect to water adsorption, deprotonation, and oxidation.^29,32,33^ Based on these observations, further structural changes can be expected upon adsorption of multiple ligands, which might eventually also lead to the stabilization of the cubic structure and a change of its activity in the water oxidation reaction. This will be subject of further investigations.

## 4 Conclusion

We have studied the interaction between manganese oxide clusters and formic acid with the use of IR-MPD spectroscopy in conjunction with DFT calculations. The IR-MPD spectra indicate binding of intact HCOOH to Mn^+^, MnO_2_^+^, and MnO_4_^+^, whereas the spectra of all other cluster sizes strongly indicate the deprotonation of formic acid. Supporting DFT calculations for three selected model systems, Mn_2_O_2_^+^, Mn_2_O_4_^+^, and Mn_4_O_4_^+^, show that depending on the cluster size the resulting formate group can bind differently. For Mn_2_O_2_^+^ formate binding in a bidentate bridging and chelating configuration are almost isoenergetic in agreement with the IR-MPD spectrum that can be best described by the co-existence of both isomers. Adding one oxygen molecule to the cluster (Mn_2_O_4_^+^) leads to the stabilization of the bidentate chelating configuration. In contrast, the tetra-manganese oxide cluster Mn_4_O_4_^+^ prefers to bind the formate group in a bidentate bridging configuration. In addition, this is found to lead to the stabilization of the 2D ring-like Mn_4_O_4_^+^ cluster core with respect to the 3D cubic geometry, alebite inducing considerable structural changes. Thus, although all Mn_*x*_O_*y*_^+^ clusters with *x* ≥ 2 are able to deprotonate formic acid the binding of the formate appears to be strongly cluster-size dependent. This finding is important with respect to the identification of suitable ligands for MnO based cluster catalysts.

## Conflicts of interest

There are no conflicts to declare.

## Supplementary Material
